# MINS-Related Risk-Factor Burden and Long-Term Outcomes After Curative Gastrectomy for Gastric Cancer

**DOI:** 10.3390/cancers18213531

**Published:** 2026-11-03

**Authors:** Yasunori Matsumoto, Shinichiro Iida, Akane Morimoto, Yuri Nishioka, Koichi Hayano, Yoshihiro Kurata, Yukiko Yamaguchi, Takeshi Toyozumi, Nobufumi Sekino, Akira Nakano, Kazuya Kinoshita, Takuma Sasaki, Michihiro Maruyama

**Affiliations:** Department of Frontier Surgery, Chiba University Graduate School of Medicine, 1-8-1 Inohana, Chuo-ku, Chiba-Shi, Chiba 260-8670, Japan

**Keywords:** gastric cancer, myocardial injury after noncardiac surgery, MINS, cardio-oncology, gastrectomy, cardiovascular risk, prognosis, overall survival

## Abstract

Background/Objectives: Cardiovascular vulnerability may influence long-term outcomes after cancer surgery, but the prognostic significance of risk factors associated with myocardial injury after noncardiac surgery (MINS) in gastric cancer is unclear. We investigated the association between MINS-related risk-factor burden and long-term outcomes after curative gastrectomy and evaluated its incremental prognostic information. Methods: We retrospectively analyzed 364 consecutive patients who underwent R0 gastrectomy for gastric adenocarcinoma between June 2016 and December 2023. Patients were categorized by MINS-related risk-factor count as 0–2, 3, or ≥4. Overall survival (OS) was the primary outcome; recurrence and cause-specific mortality were additionally evaluated using competing-risk methods. Cox regression, restricted cubic spline analysis, sensitivity analyses, and prognostic model comparisons were performed. Results: Five-year OS was 87.2%, 81.7%, and 66.6% in the 0–2, 3, and ≥4 groups, respectively (*p* < 0.001). Each additional MINS-related risk factor was associated with worse OS after adjustment for pathological stage and prognostic nutritional index (HR 1.25, 95% CI 1.08–1.46; *p* = 0.004), and the association remained consistent in expanded sensitivity models. The MINS-extended model improved overall model fit versus the base model (ΔLR χ^2^ = 8.17, *p* = 0.004; AIC 640.89 vs. 647.06), although the increase in C-index was modest (0.775 to 0.786; bootstrap 95% CI for ΔC-index, −0.005 to 0.041). Five-year cumulative incidence of non-cancer death was 5.2%, 11.9%, and 17.0% across the three groups (Gray’s *p* = 0.0098), whereas the MINS-related risk-factor group was not independently associated with gastric cancer death after adjustment for pathological stage. Conclusions: Greater MINS-related risk-factor burden was associated with poorer overall survival after curative gastrectomy, an association that appeared to be largely attributable to increased non-cancer mortality rather than gastric cancer-specific mortality. The measure overlaps substantially with conventional comorbidity and frailty indices but may provide a simple cardiovascular-oriented perspective on broader host vulnerability.

## Simple Summary

Patients with gastric cancer frequently have cardiovascular comorbidities, particularly as the population undergoing surgery becomes older. Risk factors associated with myocardial injury after noncardiac surgery (MINS) are readily available from routine preoperative information, but their relationship with long-term outcomes after curative gastrectomy remains unclear. In this retrospective cohort of 364 patients, a greater burden of MINS-related risk factors was associated with worse long-term survival and with a higher cumulative incidence of non-cancer death. The association with overall mortality remained consistent across sensitivity analyses adjusting for functional, surgical, and treatment-related factors. Benchmark analyses showed substantial overlap with conventional comorbidity and frailty measures, while gains in discrimination were modest. These findings suggest that MINS-related risk-factor burden may provide a simple cardiovascular-oriented perspective on broader host vulnerability during long-term follow-up.

## 1. Introduction

Gastric cancer (GC) remains a major global health burden and an important cause of cancer-related mortality worldwide [1,2,3]. Although advances in surgical technique, perioperative management, and multidisciplinary treatment have improved outcomes, long-term survival after gastrectomy remains strongly dependent on tumor stage, and substantial differences in prognosis persist even after curative resection [4].

Prognostic assessment in GC has traditionally focused on tumor-related characteristics. Serum tumor markers, including carcinoembryonic antigen (CEA) and carbohydrate antigen 19-9 (CA19-9), are widely used in clinical practice, but their prognostic performance is limited when used alone [5]. Host-related factors have therefore received increasing attention. Nutritional and inflammatory indices such as the prognostic nutritional index (PNI) and Glasgow Prognostic Score have been associated with survival in patients with GC [6,7,8]. Body-composition parameters, including skeletal muscle mass and adipose-tissue characteristics, have also emerged as potential prognostic markers [9,10,11]. Our group previously reported that high subcutaneous adipose-tissue radiodensity was associated with poor prognosis in patients with GC, suggesting that host tissue quality may reflect biological vulnerability [12].

In parallel, the expanding field of cardio-oncology has highlighted the interaction between cancer and cardiovascular disease and the importance of cardiovascular health throughout the cancer-care continuum [13]. This issue is particularly relevant in GC because patients undergoing gastrectomy are increasingly older and frequently have cardiovascular comorbidities. Cardiovascular vulnerability may influence not only perioperative outcomes but also long-term survival after otherwise curative cancer treatment.

Myocardial injury after noncardiac surgery (MINS), defined by postoperative cardiac troponin elevation attributable to myocardial ischemia, is associated with substantial short-term mortality after noncardiac surgery [14,15,16,17]. Large prospective studies, including the Vascular events In noncardiac Surgery patIents cOhort evaluatioN (VISION) study, identified readily ascertainable clinical characteristics associated with MINS and other perioperative cardiovascular complications [14,15,16,17]. Other established perioperative cardiovascular risk tools include the Revised Cardiac Risk Index (RCRI) and the Gupta Myocardial Infarction or Cardiac Arrest (MICA) risk calculator [18,19].

These cardiovascular risk measures were developed primarily to predict short-term perioperative cardiac events rather than long-term oncological outcomes. Whether the cumulative burden of MINS-related risk factors reflects broader host vulnerability associated with long-term survival after curative GC surgery remains unclear. Therefore, the primary objective of this study was to investigate the association between the number of MINS-related risk factors and long-term outcomes after R0 gastrectomy for GC. We further evaluated whether the MINS-related risk-factor count provided prognostic information beyond pathological stage and PNI, benchmarked its performance against age and established comorbidity/frailty measures, and examined competing non-cancer mortality and clinically apparent postoperative cardiovascular and thromboembolic events.

## 2. Materials and Methods

### 2.1. Study Design and Patients

**Figure 1:**
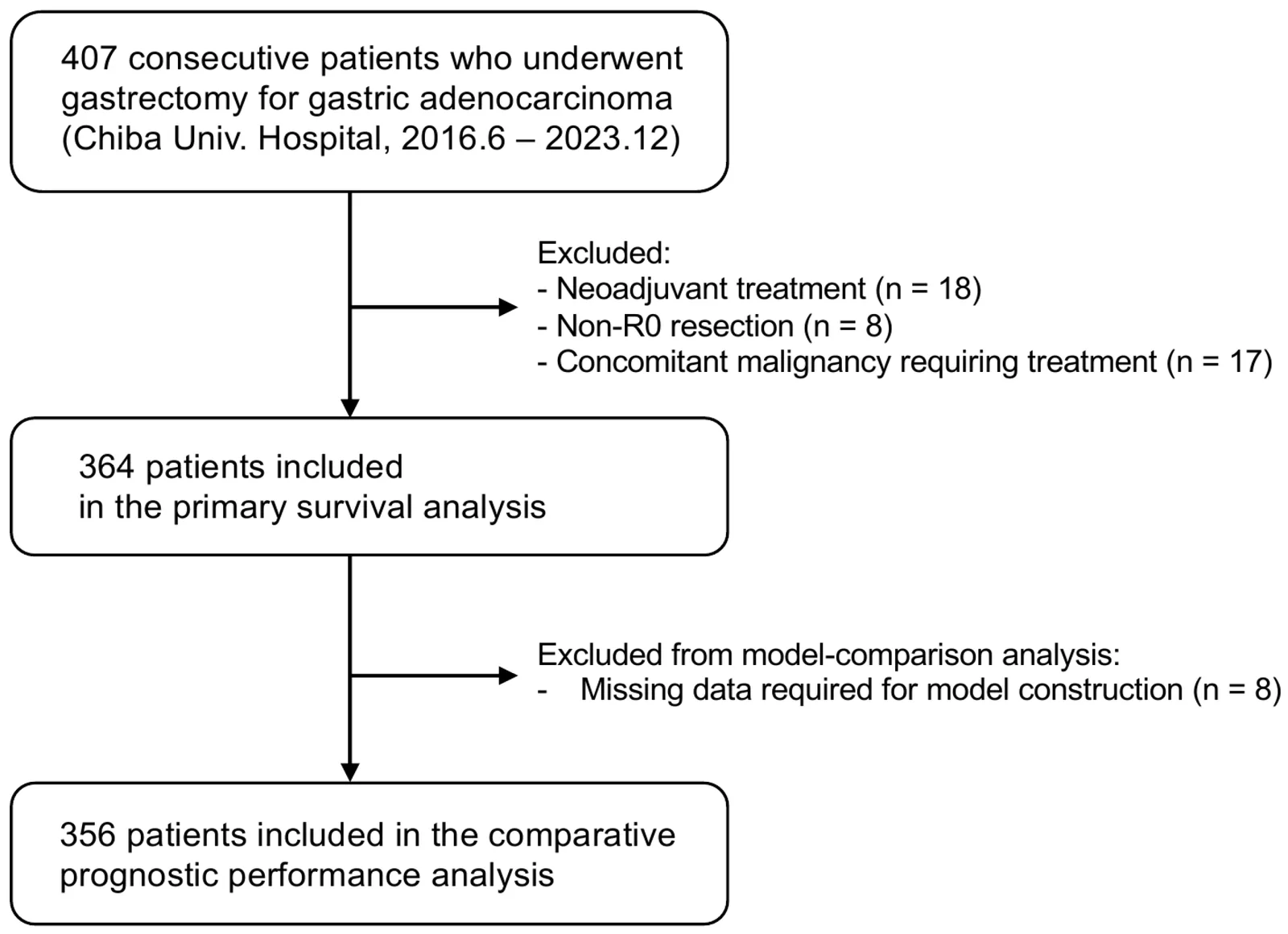
Flow diagram of patient selection. Of 407 consecutive patients who underwent gastrectomy for gastric adenocarcinoma, 364 were included in the primary survival analysis and 356 in the comparative prognostic-performance analysis.

This retrospective cohort study included consecutive patients who underwent gastrectomy for histologically confirmed gastric adenocarcinoma at Chiba University Hospital between June 2016 and December 2023. Among 407 consecutive patients, 18 who received neoadjuvant treatment, eight who underwent non-R0 resection, and 17 with concomitant malignancies requiring treatment were excluded. Consequently, 364 patients were included in the primary survival analysis. For comparative prognostic-performance analyses, eight additional patients with missing data required for model construction were excluded, leaving 356 patients (Figure 1). The database was locked in July 2026. The median follow-up period, estimated using the reverse Kaplan–Meier method, was 60 months. Because the last eligible operation was performed in December 2023, the minimum potential follow-up was approximately 30 months.

This study was approved by the Ethics Committee of the Graduate School of Medicine, Chiba University (approval no. M10866). Information regarding the study was disclosed to eligible patients, who were provided with the opportunity to opt out of participation.

### 2.2. Clinicopathological Variables

Clinical, laboratory, operative, pathological, and follow-up data were retrospectively collected from medical records. Tumors were classified according to the Japanese Classification of Gastric Carcinoma, 15th edition, and pathological stage was determined according to the eighth edition of the Union for International Cancer Control TNM classification [20,21]. Treatment was performed in accordance with the Japanese Gastric Cancer Treatment Guidelines applicable during the study period [22]. Variables included age, sex, body mass index, American Society of Anesthesiologists Physical Status (ASA-PS), Eastern Cooperative Oncology Group Performance Status (ECOG-PS), nutritional and inflammatory markers, serum CEA and CA19-9 levels, operative characteristics, postoperative complications, tumor diameter, predominant histological type, pathological stage, and receipt of adjuvant chemotherapy. Postoperative complications were classified according to the Clavien–Dindo system, and complications of grade II or higher were recorded. PNI was assessed as a host nutritional marker, and the modified Glasgow Prognostic Score (mGPS) was used as an inflammation-based index. Serum CEA and CA19-9 levels were dichotomized according to the institutional upper limits of normal for survival analyses. Clinically apparent cardiovascular and thromboembolic events occurring within 30 days after surgery were retrospectively identified when they required specialist consultation and/or treatment and were not considered directly attributable to surgical manipulation or technical complications.

### 2.3. MINS-Related Risk Factors and Comparative Clinical Indices

MINS-related risk factors were selected on the basis of clinical predictors identified in large prospective studies of myocardial injury following noncardiac surgery, particularly the VISION cohort [14]. The factors evaluated were age ≥75 years, male sex, hypertension, diabetes mellitus, atrial fibrillation, heart failure, ischemic heart disease, peripheral vascular disease, history of stroke, and renal impairment, defined as an estimated glomerular filtration rate < 60 mL/min/1.73 m^2^. The number of risk factors present in each patient was summed to generate the MINS-related risk-factor count. The observed count ranged from 0 to 7, with a median of 3 (interquartile range, 1–4). For Kaplan–Meier analyses, patients were classified into three groups: 0–2, 3, and ≥4 risk factors. For Cox regression and restricted cubic spline analyses, the count was analyzed as a continuous variable. The RCRI was calculated according to Lee et al. [18], and Gupta MICA risk according to Gupta et al. [19]. General comorbidity burden was additionally assessed using the original age-independent Charlson Comorbidity Index (CCI) [23]. The index gastric cancer was not counted as a comorbid malignancy; qualifying pre-existing non-gastric malignancies were included. Frailty was assessed using the five-item modified Frailty Index (mFI-5), comprising diabetes mellitus, chronic obstructive pulmonary disease, congestive heart failure, hypertension, and functional dependence [24]. Partial or complete functional dependence was scored as functional dependence for the mFI-5.

### 2.4. Outcomes and Follow-Up

The primary outcome was overall survival (OS), defined as the interval from gastrectomy to death from any cause or the last known follow-up. Secondary analyses evaluated recurrence and cause-specific mortality. For recurrence analyses, recurrence was treated as the event of interest and death without prior recurrence as a competing event. For cause-specific mortality, gastric cancer death and non-cancer death were treated as mutually exclusive competing events.

### 2.5. Statistical Analysis

Continuous variables are presented as medians and interquartile ranges, and categorical variables as numbers and percentages. Baseline characteristics across the three MINS-related risk-factor groups were compared using the Kruskal–Wallis test for continuous variables and the chi-square test or Fisher’s exact test, as appropriate, for categorical variables. OS curves were estimated using the Kaplan–Meier method and compared using the log-rank test; pairwise comparisons were adjusted using the Holm method. Associations with OS were evaluated using Cox proportional hazards regression. Age was modeled per 10-year increase, PNI per 5-point increase, operative time per 60-min increase, blood loss per 100 mL increase, tumor diameter per 1 cm increase, and MINS-related risk-factor burden per additional risk factor. ECOG-PS was dichotomized as 0 versus ≥1. The prespecified primary multivariable model included pathological stage, PNI, and the MINS-related risk-factor count. Given the limited number of OS events, we used expanded sensitivity models to assess robustness after additional adjustment for ECOG-PS, surgical approach, ASA-PS, blood loss, tumor diameter, or postoperative adjuvant chemotherapy, rather than indiscriminately including all covariates in one model. The proportional hazards assumption was assessed using Schoenfeld residuals. Restricted cubic spline analysis used three knots at MINS counts of 1, 3, and 5 (10th, 50th, and 90th percentiles), with a count of 3 as the reference [25,26]. For recurrence and cause-specific mortality, cumulative incidence functions were estimated using the Aalen–Johansen estimator, treating death without prior recurrence and alternative causes of death as competing events, respectively, and group differences were assessed using Gray’s test. Fine–Gray subdistribution hazard regression analysis adjusted for pathological stage was used for competing-risk analyses of cause-specific mortality. For prognostic model comparison, a base model comprising pathological stage and PNI was constructed. Extended models separately added MINS-related risk-factor count, age, RCRI, Gupta MICA risk, CCI, or mFI-5. Model fit was assessed using likelihood-ratio testing and Akaike information criterion (AIC), and discrimination using Harrell’s concordance index and time-dependent AUCs at 1, 3, and 5 years [27,28]. Ninety-five percent confidence intervals for time-dependent AUCs were estimated, and Harrell’s C-index and changes in C-index relative to the base model were internally evaluated using 1000 bootstrap resamples.

All statistical analyses were performed using R version 4.6.1 (R Foundation for Statistical Computing, Vienna, Austria), with the survival, rms, timeROC, and cmprsk packages. All tests were two-sided, and *p* < 0.05 was considered statistically significant.

## 3. Results

### 3.1. Patient Characteristics

A total of 407 consecutive patients underwent gastrectomy for GC during the study period. After exclusion of 43 patients, 364 were included in the primary analysis (Figure 1). Of these, 171 patients had 0–2 MINS-related risk factors, 90 had 3, and 103 had ≥4. The median follow-up period estimated using the reverse Kaplan–Meier method was 60 months. The median age was 73 years (IQR, 66–79), and 262 patients (72.0%) were male. The median PNI was 46.8 (IQR, 43.3–50.4). Pathological stage was I in 245 patients (67.3%), II in 70 (19.2%), and III in 49 (13.5%). Postoperative complications of Clavien-Dindo grade ≥II occurred in 96 patients (26.4%). Table 1 shows baseline clinicopathological characteristics and between-group *p*-values.

### 3.2. MINS-Related Risk-Factor Burden and Long-Term Survival

**Figure 2:**
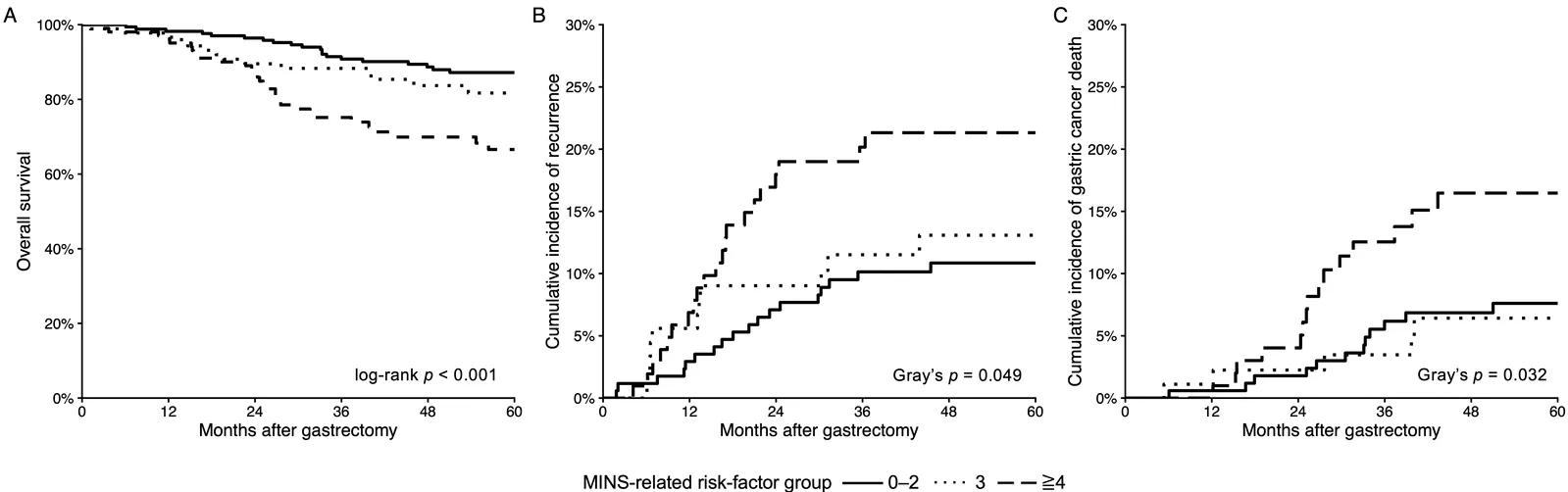
Long-term outcomes according to MINS-related risk-factor burden. (**A**) Kaplan–Meier estimates of overall survival. (**B**) Aalen–Johansen estimates of the cumulative incidence of recurrence, with death without recurrence treated as a competing event. (**C**) Aalen–Johansen estimates of the cumulative incidence of gastric cancer death, with non-cancer death treated as a competing event. Group differences were assessed using the log-rank test for overall survival and Gray’s test for competing-risk outcomes.

Kaplan–Meier analysis demonstrated significant differences in OS among the three MINS-related risk-factor groups (log-rank *p* < 0.001). Five-year OS rates were 87.2% in patients with 0–2 risk factors, 81.7% in those with 3, and 66.6% in those with ≥4 (Figure 2A). Using Aalen–Johansen estimates with death without recurrence treated as a competing event, the 5-year cumulative incidence of recurrence was 10.8%, 13.1%, and 21.3%, respectively (Gray’s *p* = 0.049; Figure 2B). The 5-year cumulative incidence of gastric cancer death was 7.6%, 6.4%, and 16.5%, respectively (Gray’s *p* = 0.032; Figure 2C), but the MINS-related risk-factor group was not independently associated with gastric cancer death after adjustment for pathological stage. During follow-up, 64 patients died: 32 from gastric cancer and 32 from non-cancer causes. The 5-year cumulative incidence of non-cancer death was 5.2%, 11.9%, and 17.0%, respectively (Gray’s *p* = 0.0098); the group with ≥4 MINS-related risk factors had a higher stage-adjusted subdistribution hazard of non-cancer death than the group with 0–2 MINS-related risk factors (sHR 3.18, 95% CI 1.37–7.39; *p* = 0.007; Supplementary Table S1). Detailed causes of non-cancer death are also provided in Supplementary Table S1.

### 3.3. Cox Proportional Hazards Analyses for Overall Survival

In univariable Cox regression, increasing age, ASA-PS 3, ECOG-PS ≥1, lower PNI, mGPS 2, minimally invasive versus open surgical approach, greater blood loss, larger tumor diameter, higher pathological stage, receipt of adjuvant chemotherapy, and greater MINS-related risk-factor burden were significantly associated with OS (Table 2). Each additional MINS-related risk factor was associated with a 34% increase in the hazard of all-cause mortality (HR 1.34, 95% CI 1.17–1.55; *p* < 0.001). In the prespecified multivariable model adjusting for pathological stage and PNI, the MINS-related risk-factor count remained associated with OS (HR 1.25 per additional factor, 95% CI 1.08–1.46; *p* = 0.004). The proportional hazards assumption was not violated for pathological stage (*p* = 0.87), PNI (*p* = 0.99), or MINS-related risk-factor count (*p* = 0.54), and the global test was non-significant (*p* = 0.96). In sensitivity analyses, the MINS association was modestly attenuated but remained directionally consistent after additional adjustment for ECOG-PS, surgical approach, ASA-PS, blood loss, or tumor diameter (HRs approximately 1.20–1.23; all *p* < 0.05; Supplementary Table S2).

### 3.4. Continuous Association Between MINS Risk-Factor Count and Mortality

**Figure 3:**
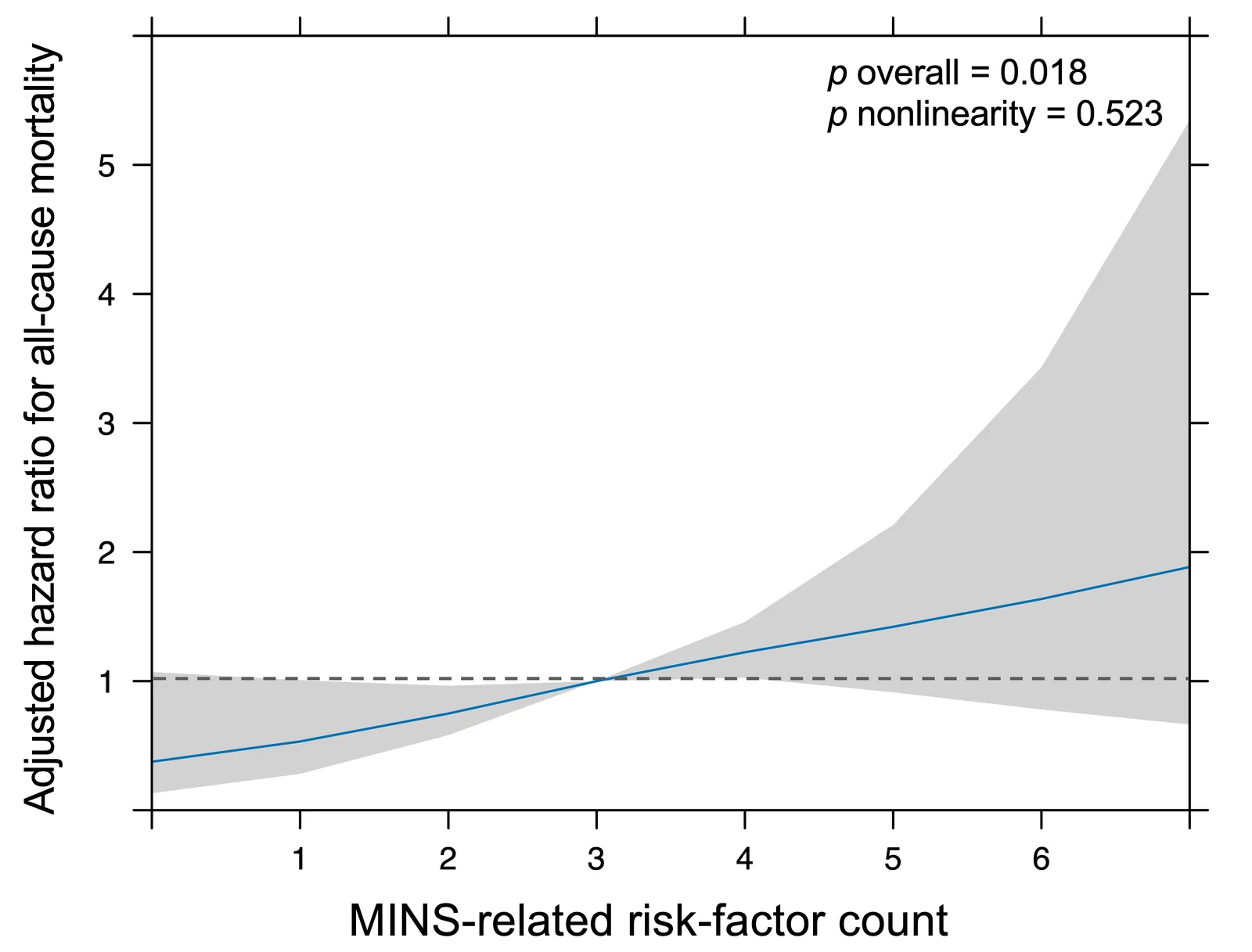
Restricted cubic spline analysis of the association between MINS-related risk-factor count and all-cause mortality. Hazard ratios were adjusted for pathological stage and PNI, with a MINS-related risk-factor count of 3 as the reference. The shaded area represents the 95% confidence interval.

In the multivariable Cox model adjusted for pathological stage and PNI, each additional MINS-related risk factor was associated with a 25% higher hazard of all-cause mortality (HR 1.25, 95% CI 1.08–1.46; *p* = 0.004). Restricted cubic spline analysis using knots at MINS counts of 1, 3, and 5 supported an overall association between increasing MINS-related risk-factor count and all-cause mortality (*p* overall = 0.018), with no evidence of nonlinearity (*p* for nonlinearity = 0.523), suggesting an approximately linear increase in mortality hazard across the observed range of MINS-related risk-factor burden (Figure 3).

### 3.5. Comparison of Prognostic Models

**Figure 4:**
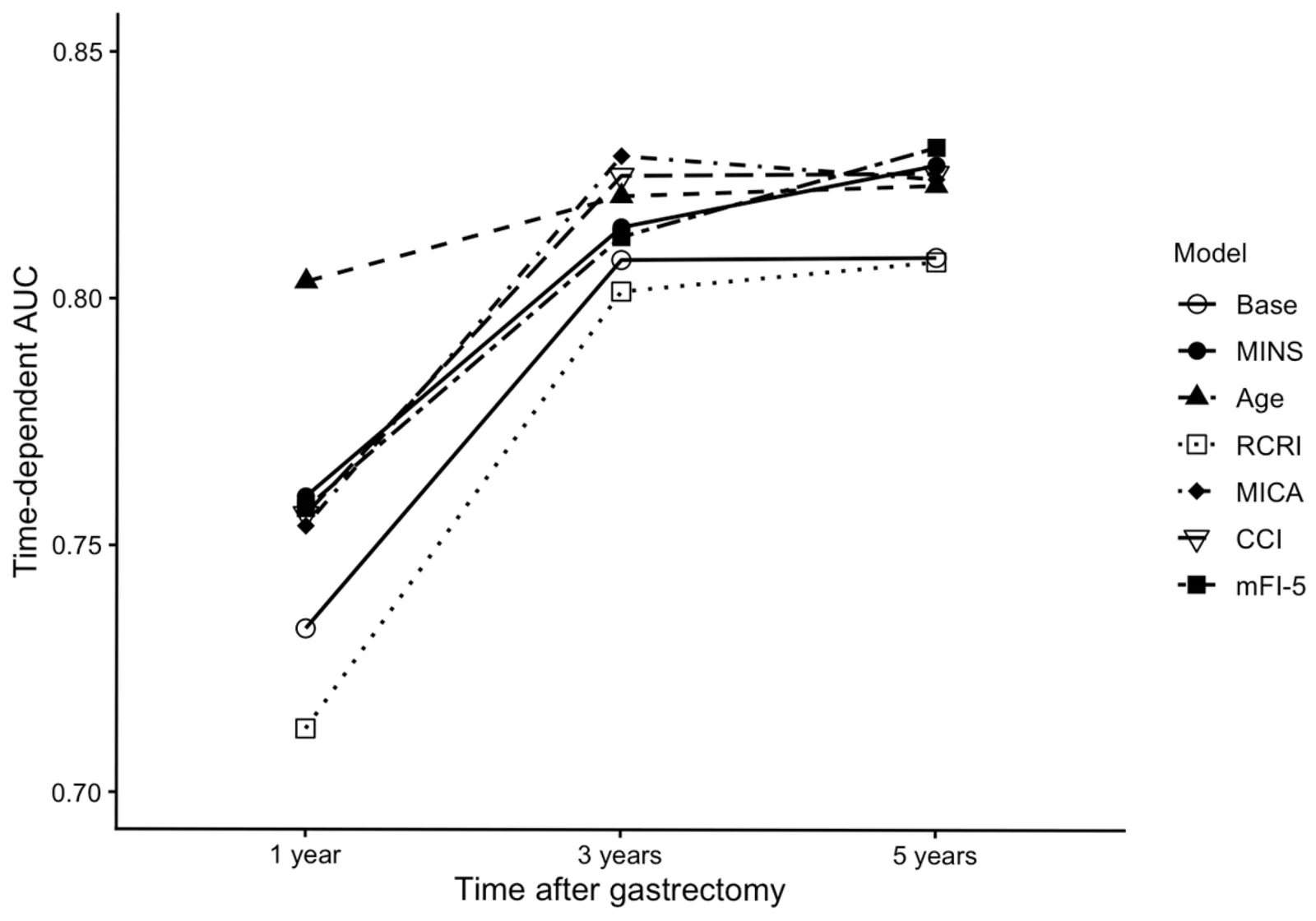
Time-dependent area under the receiver operating characteristic curve (AUC) for overall survival at 1, 3, and 5 years according to the seven prognostic models. The base model included pathological stage and prognostic nutritional index (PNI). The MINS, age, RCRI, MICA, CCI, and mFI-5 models were constructed by separately adding the MINS-related risk-factor count, age, Revised Cardiac Risk Index (RCRI), Gupta Myocardial Infarction or Cardiac Arrest (MICA) risk, Charlson Comorbidity Index (CCI), or 5-item modified Frailty Index (mFI-5), respectively, to the base model.

Among the 356 patients with complete data included in the comparative prognostic-model analysis, 62 OS events occurred. Compared with the base model comprising pathological stage and PNI, addition of the MINS-related risk-factor count significantly improved model fit (likelihood-ratio χ^2^ = 8.17, *p* = 0.004; AIC, 640.89 vs. 647.06), as did addition of age (χ^2^ = 4.67, *p* = 0.031), CCI (χ^2^ = 7.40, *p* = 0.007), and mFI-5 (χ^2^ = 6.03, *p* = 0.014). Addition of RCRI did not significantly improve model fit (χ^2^ = 1.48, *p* = 0.223), whereas MICA showed a borderline improvement (χ^2^ = 3.84, *p* = 0.050) (Table 3). The C-index of the base model was 0.775 and increased modestly to 0.786 with addition of the MINS-related risk-factor count. Corresponding C-indices were 0.786 for age, 0.771 for RCRI, 0.790 for MICA, and 0.787 for both CCI and mFI-5. Time-dependent AUCs at 1, 3, and 5 years were 0.733, 0.808, and 0.808 for the base model; 0.760, 0.815, and 0.827 for the MINS model; 0.803, 0.821, and 0.823 for the age model; 0.713, 0.802, and 0.808 for the RCRI model; 0.754, 0.829, and 0.824 for the MICA model; 0.756, 0.825, and 0.826 for the CCI model; and 0.758, 0.813, and 0.831 for the mFI-5 model (Figure 4). Overall, the models showed broadly comparable discrimination at 3 and 5 years, whereas age yielded the highest AUC at 1 year. MINS-related burden correlated with CCI and mFI-5 (Spearman’s ρ = 0.493 and 0.687, respectively). Bootstrap 95% CIs for the C-index were 0.714–0.831 for the base model and 0.731–0.840 for the MINS model; the bootstrap 95% CI for the change in C-index with MINS was −0.005 to 0.041. Full benchmark results are shown in Supplementary Table S3.

Additional analyses showed that clinically apparent cardiovascular and thromboembolic events within 30 days occurred in 12 of 364 patients (3.3%): 2/171 (1.2%) in the group with 0–2 MINS-related risk factors, 2/90 (2.2%) in the group with 3 MINS-related risk factors, and 8/103 (7.8%) in the group with ≥4 MINS-related risk factors (Fisher’s exact *p* = 0.012; Supplementary Table S4). Among patients with stage II–III disease, adjuvant chemotherapy was administered to 28/47 (59.6%), 10/32 (31.3%), and 17/40 (42.5%) patients across the three MINS-related risk-factor groups; among those for whom adjuvant therapy was guideline-recommended, the corresponding proportions were 65.8%, 37.0%, and 50.0% (*p* = 0.068; Supplementary Table S5). In exploratory sensitivity analyses additionally accounting for postoperative adjuvant chemotherapy and other clinical covariates, the MINS-related risk-factor count remained associated with OS in the full cohort (HR per factor, 1.20; 95% CI, 1.01–1.42; *p* = 0.039) and among patients with pathological stage II–III disease (HR, 1.30; 95% CI, 1.06–1.61; *p* = 0.014) (Supplementary Table S2).

## 4. Discussion

The cumulative burden of MINS-related risk factors was associated with long-term outcomes after curative gastrectomy for GC. Each additional factor remained associated with higher all-cause mortality after adjustment for pathological stage and PNI, and the association was stable across expanded sensitivity models. However, benchmark analyses also showed substantial overlap with general comorbidity and frailty, and discrimination gains were modest. Thus, these findings support MINS-related risk-factor burden as a marker of broader host vulnerability rather than a disease-specific or independently validated prognostic score.

These findings fit within the broader cardio-oncology concept that long-term outcome reflects interactions among cancer, treatment, pre-existing cardiovascular disease, and physiological reserve [13]. This is particularly relevant in patients undergoing gastrectomy, who are increasingly older and frequently have cardiovascular comorbidities. Cardiovascular vulnerability may therefore influence outcomes beyond the immediate perioperative period.

MINS provides a clinically relevant framework for examining this relationship, but the distinction between actual postoperative MINS and preoperative MINS-related risk factors is important. VISION studies established the association between postoperative troponin elevation and short-term mortality and identified clinical characteristics associated with perioperative myocardial injury [14,15,16,17]. In the present study, we did not perform routine postoperative troponin surveillance; therefore, we could not ascertain actual MINS, particularly asymptomatic myocardial injury. Consequently, the MINS-related risk-factor count should be interpreted as a pragmatic measure of cardiovascular and systemic vulnerability rather than a diagnostic measure of MINS.

Our previous study suggested that assessment for postoperative myocardial injury after GC surgery may contribute to early detection of postoperative complications [29]. Postoperative complications, particularly infectious complications and anastomotic leakage, have been associated with poorer long-term survival after gastrectomy [30,31,32,33], suggesting that perioperative vulnerability may have implications beyond the immediate postoperative period. However, in the present cohort, Clavien–Dindo grade ≥II complications were not significantly associated with OS (HR 1.31, 95% CI 0.78–2.21; *p* = 0.305). In contrast, competing-risk analyses showed that the cumulative incidence of non-cancer death increased across MINS-related risk-factor groups, with a significantly higher stage-adjusted subdistribution hazard in the group with ≥4 MINS-related risk factors. Although the cumulative incidence of gastric cancer death differed among the groups in unadjusted analysis, the MINS-related risk-factor group was not independently associated with gastric cancer death after adjustment for pathological stage. These findings suggest that the association between MINS-related risk-factor burden and OS appears to be driven largely by non-cancer mortality and broader host vulnerability rather than by an independent association with gastric cancer-specific mortality. Clinically apparent cardiovascular and thromboembolic events within 30 days were also more frequent in the highest MINS-related risk-factor group, although only 12 such events occurred and no multivariable analysis was attempted.

Surgical approach and intraoperative blood loss require cautious interpretation. Patients with greater MINS-related risk-factor burden more often underwent open surgery and had greater blood loss. In clinical practice, surgeons selected the approach based on patient and tumor characteristics; in some higher-risk patients, they may have chosen open surgery when shorter operative time was prioritized or when the physiological effects of pneumoperitoneum were considered undesirable. Thus, the observed association is susceptible to confounding by indication and treatment-selection bias. Blood loss was also strongly related to surgical approach. Importantly, sensitivity analyses adjusting for approach and other perioperative factors yielded similar MINS estimates, but the present data do not support preferential selection of open gastrectomy for patients with high MINS-related risk-factor burden. Antithrombotic medication use also increased with MINS-related risk-factor burden (Supplementary Table S6). Greater intraoperative blood loss has also been associated with unfavorable long-term outcomes after gastrectomy [34,35].

Postoperative adjuvant chemotherapy represents another potential pathway linking preoperative vulnerability with long-term outcome. Among patients with pathological stage II–III disease, adjuvant chemotherapy was administered to 59.6%, 31.3%, and 42.5% of patients in the MINS 0–2, 3, and ≥4 groups, respectively. Although these proportions did not decrease monotonically with increasing MINS-related burden, the numerical differences suggest that treatment exposure may have varied by postoperative fitness, comorbidity, or other clinical factors. In exploratory sensitivity analyses additionally accounting for adjuvant chemotherapy and other clinical covariates, the MINS-related risk-factor count remained associated with OS in both the full cohort and patients with stage II–III disease. Nevertheless, adjuvant chemotherapy is a postoperatively determined treatment variable and may partly lie on the causal pathway between patient vulnerability and long-term outcome. Differential treatment exposure may therefore have contributed to the observed survival differences.

The MINS-related risk-factor burden overlaps with established host-related prognostic constructs. CCI and mFI-5 were correlated with MINS burden and were themselves associated with OS when added to the same oncological-nutritional base model. Discrimination was broadly similar among the MINS-, age-, CCI-, and mFI-5-extended models, and bootstrap confidence intervals for changes in C-index included zero. These results do not establish superiority of the MINS-based measure. Rather, they suggest that cardiovascular risk-factor burden captures prognostic information partly shared with multimorbidity and frailty and not explained by chronological age alone.

Model comparison should therefore be interpreted primarily in terms of incremental model fit rather than a large improvement in discrimination. The MINS-extended model showed the largest likelihood-ratio improvement and lowest AIC among the evaluated age/comorbidity/frailty extensions, but absolute differences in C-index and time-dependent AUC were small. At 1 year, age showed numerically higher discrimination, while CCI or mFI-5 performed similarly or slightly better at some later time points. These findings support a complementary rather than replacement role for MINS-related risk-factor burden.

The potential clinical value of the MINS-related risk-factor count lies in risk awareness rather than treatment exclusion. Even in the group with ≥4 MINS-related risk factors, 66.6% of patients were alive at 5 years, and this study does not support denying curative surgery or selecting a specific operative approach based on this count. Instead, a high burden may identify patients who warrant closer attention to cardiovascular, renal, respiratory, nutritional, and functional health during postoperative follow-up and may provide additional context for discussions of long-term prognosis with patients and families. Whether such risk recognition can improve outcomes through targeted surveillance or intervention requires prospective study.

This study has limitations. Its retrospective single-center design introduces potential selection bias and residual confounding, and the limited number of deaths constrained multivariable model complexity; a parsimonious primary model was therefore supplemented by expanded sensitivity analyses. The MINS-related risk-factor count assigns equal weight to factors with potentially different biological effects and substantially overlaps with conventional comorbidity and frailty measures. Routine postoperative troponin surveillance was not performed, and asymptomatic myocardial injury could not be systematically identified; therefore, the true incidence of MINS was unknown. Postoperative cardiovascular and thromboembolic events were identified retrospectively only when clinically apparent and requiring specialist consultation and/or treatment. Cause-of-death classification was also retrospective, and some non-cancer deaths remained of unknown cause. Residual confounding and potential mediation by differential postoperative treatment exposure, including adjuvant chemotherapy, cannot be excluded. Generalizability is limited because this was a Japanese cohort with relatively low BMI, generally preserved performance status, and a treatment pathway dominated by upfront gastrectomy; contemporary Western cohorts and patients receiving perioperative systemic therapy may differ substantially. These factors should be considered when interpreting the applicability of the findings to other treatment settings.

Strengths include a consecutive R0-resection cohort, clearly defined exclusions, and mature follow-up (reverse Kaplan–Meier median, 60 months; minimum potential follow-up, approximately 30 months). The association was examined using complementary methods, including Kaplan–Meier analysis, multivariable Cox regression, restricted cubic splines, Aalen–Johansen cumulative-incidence estimation, competing-risk regression, sensitivity models, model-fit statistics, C-index, time-dependent AUCs, and benchmark comparisons with established comorbidity and frailty measures.

## 5. Conclusions

MINS-related risk-factor burden was associated with poorer overall survival after curative gastrectomy, an association that appeared to be largely attributable to increased non-cancer mortality rather than gastric cancer-specific mortality. The measure overlaps substantially with conventional comorbidity and frailty indices but may provide a simple cardiovascular-oriented perspective on broader host vulnerability.

## Figures and Tables

**Table 1:** Clinicopathological characteristics of the study population according to the MINS-related risk-factor count.

Characteristics	Overall (n = 364)	MINS 0–2 (n = 171)	MINS 3 (n = 90)	MINS ≥ 4 (n = 103)	*p*-Value
Age, years, median [IQR]	73 [66–79]	68 [60–73]	74 [69–79]	79 [75–82]	<0.001
Sex, male/female, n	262/102	97/74	72/18	93/10	<0.001
BMI, kg/m^2^, median [IQR]	23.2 [21.2–25.5]	22.7 [20.3–24.6]	24.0 [22.2–26.1]	23.6 [21.8–25.5]	<0.001
ASA-PS, 1/2/3, n	40/254/70	35/122/14	4/64/22	1/68/34	<0.001
ECOG-PS, 0/1/2/3, n	228/120/13/3	129/37/5/0	55/32/2/1	44/51/6/2	<0.001
**MINS-related risk factors, n (%)**					—
Age ≥75 years	152 (41.8)	31 (18.1)	41 (45.6)	80 (77.7)	<0.001
Male sex	262 (72.0)	97 (56.7)	72 (80.0)	93 (90.3)	<0.001
Hypertension	210 (57.7)	54 (31.6)	69 (76.7)	87 (84.5)	<0.001
Diabetes mellitus	75 (20.6)	5 (2.9)	27 (30.0)	43 (41.8)	<0.001
Atrial fibrillation	23 (6.3)	1 (0.6)	5 (5.6)	17 (16.5)	<0.001
Heart failure	18 (5.0)	0 (0)	2 (2.2)	16 (15.5)	<0.001
Ischemic heart disease	39 (10.7)	2 (1.2)	7 (7.8)	30 (29.1)	<0.001
Peripheral vascular disease	18 (5.0)	0 (0)	3 (3.3)	15 (14.6)	<0.001
History of stroke	32 (8.8)	2 (1.2)	4 (4.4)	26 (25.2)	<0.001
Renal impairment (eGFR < 60 mL/min/1.73 m^2^)	128 (35.2)	11 (6.4)	40 (44.4)	77 (74.8)	<0.001
**Cardiovascular risk indices**					—
RCRI, 1/2/≥3, n	271/70/23	163/7/1	68/20/2	40/43/20	<0.001
Gupta MICA, %, median [IQR]	0.3 [0.3–0.4]	0.3 [0.2–0.3]	0.4 [0.3–0.9]	0.4 [0.4–1.4]	<0.001
**Nutritional, inflammatory, and serum markers**					—
PNI, median [IQR]	46.8 [43.3–50.4]	47.6 [44.5–51.3]	46.0 [42.5–50.6]	45.8 [41.4–49.1]	<0.001
mGPS, 0/1/2, n	339/10/13	163/5/3	83/2/4	93/3/6	0.429
CEA, ng/mL, median [IQR]	2.3 [1.5–3.3]	2.0 [1.3–2.9]	2.6 [1.5–3.8]	2.4 [1.8–3.3]	<0.001
CA19-9, U/mL, median [IQR]	10.9 [6.0–19.3]	10.0 [5.4–18.1]	12.1 [8.1–19.2]	13.0 [5.3–23.8]	0.162
**Surgical approach, n (%)**					<0.001
Open	136 (37.4)	47 (27.5)	38 (42.2)	51 (49.5)	
Minimally invasive	228 (62.6)	124 (72.5)	52 (57.8)	52 (50.5)	
**Type of gastrectomy, n (%)**					0.770
DG	257 (70.6)	122 (71.4)	65 (72.2)	70 (68.0)	
TG	63 (17.3)	28 (16.4)	16 (17.8)	19 (18.5)	
PG	29 (8.0)	16 (9.4)	6 (6.7)	7 (6.8)	
Other	15 (4.1)	5 (2.9)	3 (3.3)	7 (6.8)	
Operative time, min, median [IQR]	297 [248–362]	292 [245–361]	302 [255–374]	298 [247–358]	0.706
Blood loss, mL, median [IQR]	90 [5–270]	50 [5–150]	100 [10–400]	150 [10–423]	<0.001
Postoperative complications (CD grade ≥II), n (%)	96 (26.4)	42 (24.6)	21 (23.3)	33 (32.0)	0.298
Postoperative hospital stay, days, median [IQR]	11 [9–15]	11 [9–15]	11 [9–14]	12 [10–19]	0.003
Tumor diameter, cm, median [IQR]	3.0 [2.0–4.7]	2.5 [1.7–4.0]	3.5 [2.2–5.2]	3.2 [2.1–4.8]	0.003
**Predominant histological type, n (%)**					<0.001
Differentiated	207 (56.9)	76 (44.4)	63 (70.0)	68 (66.0)	
Undifferentiated	133 (36.5)	81 (47.4)	22 (24.4)	30 (29.1)	
Other/unknown	24 (6.6)	14 (8.2)	5 (5.6)	5 (4.9)	
**pStage, n (%)**					0.327
I	245 (67.3)	124 (72.5)	58 (64.4)	63 (61.2)	
II	70 (19.2)	29 (17.0)	19 (21.1)	22 (21.4)	
III	49 (13.5)	18 (10.5)	13 (14.4)	18 (17.5)	
Adjuvant chemotherapy, n (%)	55 (15.1)	28 (16.4)	10 (11.1)	17 (16.5)	0.474

Continuous variables are presented as medians [interquartile ranges], and categorical variables as numbers (percentages). Continuous variables were compared using the Kruskal–Wallis test, and categorical variables using the Pearson chi-square test or Fisher’s exact test, as appropriate. Percentages may not total 100% because of rounding. Data were missing for mGPS in two patients. Abbreviations: IQR—interquartile range; BMI—body mass index; ASA-PS—American Society of Anesthesiologists Physical Status; ECOG-PS—Eastern Cooperative Oncology Group Performance Status; MINS—myocardial injury after noncardiac surgery; eGFR—estimated glomerular filtration rate; RCRI—Revised Cardiac Risk Index; MICA—myocardial infarction or cardiac arrest; PNI—prognostic nutritional index; mGPS—modified Glasgow Prognostic Score; CEA—carcinoembryonic antigen; CA19-9—carbohydrate antigen 19-9; DG—distal gastrectomy; TG—total gastrectomy; PG—proximal gastrectomy; CD—Clavien–Dindo.

**Table 2:** Univariable and multivariable Cox proportional hazards analyses for overall survival.

Variable	Univariable HR (95% CI)	*p*-Value	Multivariable HR (95% CI)	*p*-Value
Age, per 10-year increase	1.68 (1.25–2.24)	<0.001	—	—
Sex, male vs. female	1.40 (0.77–2.53)	0.265	—	—
BMI, per 1 kg/m^2^ increase	1.00 (0.93–1.07)	0.909	—	—
**ASA-PS**				
2 vs. 1	2.51 (0.78–8.11)	0.123	—	—
3 vs. 1	4.63 (1.37–15.68)	0.014	—	—
ECOG-PS, ≥1 vs. 0	3.57 (2.14–5.95)	<0.001	—	—
PNI, per 5-point increase	0.63 (0.52–0.77)	<0.001	0.78 (0.63–0.97)	0.024
**mGPS**				
1 vs. 0	1.87 (0.59–6.00)	0.289	—	—
2 vs. 0	3.63 (1.56–8.45)	0.003	—	—
CEA, elevated vs. normal	1.89 (0.96–3.72)	0.064	—	—
CA19-9, elevated vs. normal	1.86 (0.91–3.76)	0.087	—	—
Surgical approach, MIS vs. open	0.18 (0.10–0.31)	<0.001	—	—
**Type of gastrectomy**				
TG vs. DG	1.69 (0.95–3.01)	0.076	—	—
PG vs. DG	0.86 (0.31–2.41)	0.779	—	—
Other vs. DG	1.39 (0.43–4.48)	0.583	—	—
Operative time, per 60-min increase	0.89 (0.75–1.06)	0.181	—	—
Blood loss, per 100-mL increase	1.10 (1.05–1.14)	<0.001	—	—
Postoperative complications, yes vs. no	1.31 (0.78–2.21)	0.305	—	—
Tumor diameter, per 1-cm increase	1.16 (1.09–1.23)	<0.001	—	—
**Predominant histological type**				
Undifferentiated vs. differentiated	1.60 (0.97–2.61)	0.064	—	—
Other/unknown vs. differentiated	0.26 (0.03–1.88)	0.180	—	—
**pStage (reference: stage I)**				
II vs. I	3.51 (1.84–6.70)	<0.001	3.28 (1.71–6.26)	<0.001
III vs. I	10.30 (5.73–18.38)	<0.001	7.23 (3.90–13.41)	<0.001
Adjuvant chemotherapy, yes vs. no	2.72 (1.60–4.61)	<0.001	—	—
MINS-related risk-factor count, per additional factor	1.34 (1.17–1.55)	<0.001	1.25 (1.08–1.46)	0.004

Hazard ratios (HRs) are presented with 95% confidence intervals (CIs). The multivariable model included pathological stage (pStage), prognostic nutritional index (PNI), and the MINS-related risk-factor count. CEA and CA19-9 were dichotomized according to the institutional upper limits of normal. Abbreviations: ASA-PS, American Society of Anesthesiologists Physical Status; ECOG-PS, Eastern Cooperative Oncology Group Performance Status; PNI, prognostic nutritional index; mGPS, modified Glasgow Prognostic Score; CEA, carcinoembryonic antigen; CA19-9, carbohydrate antigen 19-9; MIS, minimally invasive surgery; DG, distal gastrectomy; TG, total gastrectomy; PG, proximal gastrectomy; MINS, myocardial injury after noncardiac surgery.

**Table 3:** Comparison of prognostic performance among the seven models.

Model	ΔLR χ^2^	*p-* Value	AIC	C-Index	1-Year AUC	3-Year AUC	5-Year AUC
Base	—	—	647.06	0.775	0.733	0.808	0.808
MINS	8.17	0.004	640.89	0.786	0.760	0.815	0.827
Age	4.67	0.031	644.39	0.786	0.803	0.821	0.823
RCRI	1.48	0.223	647.58	0.771	0.713	0.802	0.808
MICA	3.84	0.050	645.22	0.790	0.754	0.829	0.824
CCI	7.40	0.007	641.67	0.787	0.756	0.825	0.826
mFI-5	6.03	0.014	643.03	0.787	0.758	0.813	0.831

Data are presented for 356 patients with complete data included in the comparative prognostic-model analysis, among whom 62 overall-survival events occurred. The base model included pathological stage (pStage) and prognostic nutritional index (PNI). The MINS, age, RCRI, MICA, CCI, and mFI-5 models were constructed by separately adding the MINS-related risk-factor count, age, Revised Cardiac Risk Index (RCRI), Gupta Myocardial Infarction or Cardiac Arrest (MICA) risk, Charlson Comorbidity Index (CCI), or 5-item modified Frailty Index (mFI-5), respectively, to the base model. ΔLR χ^2^ represents the change in the likelihood-ratio chi-square statistic relative to the base model. AIC—Akaike information criterion; AUC—area under the curve; MINS—myocardial injury after noncardiac surgery.

## Data Availability

The data presented in this study are available from the corresponding author upon reasonable request. The data are not publicly available due to privacy and ethical restrictions.

## Supplementary Materials

The following supporting information can be downloaded at: https://www.mdpi.com/article/10.3390/cancers18213531/s1, Table S1: Causes of death and competing-risk analyses according to MINS-related risk-factor burden; Table S2: Sensitivity analyses for the association between MINS-related risk-factor burden and overall survival; Table S3: Benchmark comparison of prognostic models incorporating MINS-related risk-factor burden, age, CCI, and mFI-5; Table S4: Clinically apparent cardiovascular and thromboembolic events within 30 days after gastrectomy; Table S5: Postoperative adjuvant chemotherapy according to MINS-related risk-factor burden; Table S6: Antithrombotic medication use according to MINS-related risk-factor burden.
