# Supplementary material for: MINS-Related Risk-Factor Burden and Long-Term Outcomes After Curative Gastrectomy for Gastric Cancer
Source: Cancers (Basel). 2026 Nov 3;18(21):3531. doi: 10.3390/cancers18213531 (PMC13762433; doi:10.3390/cancers18213531)
Supplement: Supplementary file 1 [file cancers-18-03531-s001.zip › cancers-4560462-supplementary.pdf]

Supplementary Materials

MINS-Related Risk-Factor Burden and Long-Term Outcomes After Curative Gastrectomy for Gastric Cancer

Manuscript ID: cancers-4560462

Supplementary Table S1.

Causes of death and competing-risk analyses according to MINS-related risk-factor burden

A. Causes of death

Outcome/cause	MINS 0–2 (n = 171)	MINS 3 (n = 90)	MINS ≥4 (n = 103)	Total (n = 364)
Alive/censored	151	76	73	300
Gastric cancer death	12	5	15	32
Non-cancer death, total	8	9	15	32
Cardiovascular	0	1	3	4
Cerebrovascular	0	1	1	2
Respiratory	3	1	4	8
Infection	0	1	0	1
Other malignancy	2	1	3	6
Other	2	1	0	3
Unknown	1	3	4	8
All deaths	20	14	30	64

B. Five-year cumulative incidence

Competing-risk outcome	MINS 0–2	MINS 3	MINS ≥4	Gray's test P value
Gastric cancer death	7.6%	6.4%	16.5%	0.032
Non-cancer death	5.2%	11.9%	17.0%	0.0098

C. Stage-adjusted Fine–Gray subdistribution hazard models

Outcome	Comparison	sHR (95% CI)	P value
Gastric cancer death	MINS 3 vs. 0–2	0.61 (0.21–1.77)	0.360
Gastric cancer death	MINS ≥4 vs. 0–2	1.53 (0.72–3.26)	0.270
Gastric cancer death	pStage II vs. I	5.39 (1.70–17.05)	0.004
Gastric cancer death	pStage III vs. I	25.91 (9.56–70.22)	<0.001
Non-cancer death	MINS 3 vs. 0–2	2.26 (0.89–5.78)	0.088
Non-cancer death	MINS ≥4 vs. 0–2	3.18 (1.37–7.39)	0.007
Non-cancer death	pStage II vs. I	2.38 (1.09–5.23)	0.031
Non-cancer death	pStage III vs. I	2.18 (0.89–5.33)	0.087

For gastric cancer death, non-cancer death was treated as the competing event; for non-cancer death, gastric cancer death was treated as the competing event. Fine–Gray models were adjusted for pathological stage. sHR, subdistribution hazard ratio; CI, confidence interval; MINS, myocardial injury after noncardiac surgery.

Supplementary Table S2. Sensitivity analyses for the association between MINS-related risk-factor burden and overall survival

Model	n	Events	MINS HR per additional factor (95% CI)	P value
Primary: pStage + PNI + MINS	356	62	1.25 (1.08–1.46)	0.004
+ ECOG-PS	356	62	1.23 (1.05–1.43)	0.011
+ Surgical approach	356	62	1.23 (1.05–1.43)	0.009
+ ECOG-PS + surgical approach	356	62	1.21 (1.03–1.41)	0.019
+ ECOG-PS + surgical approach + ASA-PS	356	62	1.21 (1.02–1.43)	0.025
+ ECOG-PS + surgical approach + blood loss	355	62	1.21 (1.03–1.41)	0.022
+ ECOG-PS + surgical approach + tumor size	345	62	1.20 (1.03–1.40)	0.023
+ ECOG-PS + surgical approach + ASA-PS + postoperative adjuvant chemotherapy	356	62	1.20 (1.01–1.42)	0.039
Stage II–III subset: + ECOG-PS + surgical approach + ASA-PS + postoperative adjuvant chemotherapy	116	42	1.30 (1.06–1.61)	0.014

All models included pathological stage and PNI. ECOG-PS was dichotomized as 0 versus ≥1. Surgical approach was modeled as minimally invasive versus open surgery. ASA-PS was modeled categorically. Blood loss was modeled per 100-mL increase and tumor size per 1-cm increase. The exploratory adjuvant-chemotherapy models additionally included ECOG-PS, surgical approach, ASA-PS, and postoperative adjuvant chemotherapy; the stage II–III subset model compared pathological stage III with stage II. Because postoperative adjuvant chemotherapy is determined after surgery and may partly lie on the causal pathway between patient vulnerability and long-term outcome, these analyses were considered supportive rather than primary. Differences in n reflect missing covariate data. HR, hazard ratio; CI, confidence interval; PNI, prognostic nutritional index; ECOG-PS, Eastern Cooperative Oncology Group Performance Status; ASA-PS, American Society of Anesthesiologists Physical Status; MINS, myocardial injury after noncardiac surgery.

Supplementary Table S3. Benchmark comparison of prognostic models incorporating MINS-related risk-factor burden, age, CCI, and mFI-5

A. Overall model fit and Harrell's C-index

Model	ΔLR χ^2 vs. base	P value	AIC	C-index (bootstrap 95% CI)	ΔC-index vs. base (bootstrap 95% CI)
Base	—	—	647.06	0.775 (0.714–0.831)	—
+ MINS	8.17	0.004	640.89	0.786 (0.731–0.840)	+0.010 (−0.005 to 0.041)
+ Age	4.67	0.031	644.39	0.786 (0.730–0.840)	+0.011 (−0.005 to 0.041)
+ CCI	7.40	0.007	641.67	0.787 (0.735–0.841)	+0.012 (−0.007 to 0.044)
+ mFI-5	6.03	0.014	643.03	0.787 (0.734–0.840)	+0.012 (−0.004 to 0.046)

B. Time-dependent AUCs

Model	1-year AUC (95% CI)	3-year AUC (95% CI)	5-year AUC (95% CI)
Base	0.733 (0.566–0.900)	0.808 (0.742–0.874)	0.808 (0.746–0.871)
+ MINS	0.760 (0.613–0.907)	0.815 (0.752–0.877)	0.827 (0.769–0.886)
+ Age	0.803 (0.703–0.904)	0.821 (0.759–0.883)	0.823 (0.762–0.884)
+ CCI	0.756 (0.582–0.930)	0.825 (0.765–0.885)	0.826 (0.766–0.885)
+ mFI-5	0.758 (0.613–0.902)	0.813 (0.751–0.874)	0.831 (0.775–0.887)

All models included 356 patients with 62 deaths. The base model comprised pathological stage and PNI. Extended models separately added the MINS-related risk-factor count, continuous age, the age-independent Charlson Comorbidity Index (CCI), or the 5-item modified Frailty Index (mFI-5). Likelihood-ratio tests compare each extended model with the base model. C-index confidence intervals and changes in C-index were estimated using 1,000 bootstrap resamples. Time-dependent AUC confidence intervals were estimated using the timeROC framework. AIC, Akaike information criterion; AUC, area under the curve; CI, confidence interval; LR, likelihood ratio; PNI, prognostic nutritional index.

Supplementary Table S4. Clinically apparent cardiovascular and thromboembolic events within 30 days after gastrectomy

Event	MINS 0–2 (n = 171)	MINS 3 (n = 90)	MINS ≥4 (n = 103)	Total (n = 364)	P value
Any cardiovascular/thromboembolic event, n (%)	2 (1.2)	2 (2.2)	8 (7.8)	12 (3.3)	0.012
Pulmonary thromboembolism	1	1	3	5	—
Heart failure	0	0	3	3	—
Coronary vasospastic angina	1	0	0	1	—
Cardiac tamponade	0	0	1	1	—
Cerebral infarction	0	0	1	1	—
Splenic infarction	0	1	0	1	—

Events were retrospectively defined as clinically apparent cardiovascular or thromboembolic events occurring within 30 days that required specialist consultation and/or treatment and were not considered directly attributable to surgical manipulation or technical complications. The overall between-group comparison was performed using Fisher's exact test. Routine postoperative troponin surveillance was not performed; therefore, asymptomatic myocardial injury and the true incidence of MINS could not be determined. Detailed event subtypes are presented descriptively without separate hypothesis testing. MINS, myocardial injury after noncardiac surgery.

Supplementary Table S5. Postoperative adjuvant chemotherapy according to MINS-related risk-factor burden

A. Patients with pathological stage II–III disease

Variable	MINS 0–2	MINS 3	MINS ≥4	Total
Stage II–III patients, n	47	32	40	119
Adjuvant chemotherapy received, n (%)	28 (59.6)	10 (31.3)	17 (42.5)	55 (46.2)
Adjuvant chemotherapy not received, n (%)	19 (40.4)	22 (68.8)	23 (57.5)	64 (53.8)

B. Patients for whom postoperative adjuvant chemotherapy was guideline-recommended

Variable	MINS 0–2	MINS 3	MINS ≥4	Total
Guideline-recommended patients, n	38	27	34	99
Adjuvant chemotherapy received, n (%)	25 (65.8)	10 (37.0)	17 (50.0)	52 (52.5)
Adjuvant chemotherapy not received, n (%)	13 (34.2)	17 (63.0)	17 (50.0)	47 (47.5)

Among patients for whom postoperative adjuvant chemotherapy was guideline-recommended, the between-group difference in treatment receipt did not reach statistical significance (Pearson’s chi-square test, $P = 0.068$). MINS, myocardial injury after noncardiac surgery.

Supplementary Table S6. Antithrombotic medication use according to MINS-related risk-factor burden

A. Antithrombotic medication use by MINS-related risk-factor group

Variable	MINS 0–2 (n = 171)	MINS 3 (n = 90)	MINS ≥4 (n = 103)	P value
Antithrombotic medication, n (%)	5 (2.9)	20 (22.2)	60 (58.3)	<0.001

B. Exploratory comparison of intraoperative blood loss according to antithrombotic medication use

Antithrombotic medication	n	Blood loss, mL median (IQR)	P value
No	279	75 (5–210)	0.068
Yes	85	140 (10–330)	

Data are presented as n (%) or median (interquartile range [IQR]). Antithrombotic medication use across MINS-related risk-factor groups was compared using Pearson’s chi-square test. Blood loss according to antithrombotic medication use was compared using the Wilcoxon rank-sum test. MINS, myocardial injury after noncardiac surgery.
